# Impact of a dietary intervention with French maritime pine bark extract (Pycnogenol^®^) on bone turnover markers in women with osteoporotic bone loss: a randomized, placebo-controlled double-blind study—Part I

**DOI:** 10.3389/fnut.2026.1923949

**Published:** 2026-10-12

**Authors:** Christian Scherg, Franca Genest, Jonas Maghary, Lothar Seefried, Denitsa Docheva, Markus Krischke, Petra Högger

**Affiliations:** 1 Institute of Pharmacy and Food Chemistry, University of Würzburg, Würzburg, Germany; 2 Osteology/Clinical Trial Unit, University of Würzburg, Würzburg, Germany; 3 Department of Musculoskeletal Tissue Regeneration, Orthopaedic Hospital König-Ludwig-Haus, University of Würzburg, Würzburg, Germany; 4 Julius-von-Sachs-Institute of Biosciences, Biocenter, Pharmaceutical Biology, University of Würzburg, Würzburg, Germany

**Keywords:** bone resorption markers, bone turnover, dietary supplementation, human mesenchymal stromal cells, osteogenic differentiation, osteoporosis, Pycnogenol^®^

## Abstract

**Introduction:**

Osteoporosis is a highly prevalent health concern both in terms of disability to an individual and costs to society. While antiresorptive drugs are the standard treatment, nutritional supplementation may offer additional advantages, particularly in the pre-therapeutic stage of osteopenia or clinically stable osteoporosis. The French maritime pine bark extract Pycnogenol^®^ exhibits antioxidant and anti-inflammatory properties, but its influence on bone turnover has not been investigated in humans yet.

**Methods:**

In this randomized, placebo-controlled, double-blind clinical trial, 67 women received either Pycnogenol^®^ (100 mg twice daily; *n* = 33) or placebo (*n* = 34) during a 3-month placebo-controlled phase, followed by a 3-month active-treatment extension, in which all participants took Pycnogenol^®^. The primary endpoint included changes in the bone resorption marker C-terminal telopeptide of type I collagen (CTX-1) and bone formation marker procollagen type I N propeptide (P1NP) in serum. Secondary endpoints comprised other bone turnover markers (N-terminal telopeptide of type I collagen, NTX-1; tartrate-resistant acid phosphatase 5b, TRAP5b; bone sialoprotein, BSP), an inflammatory marker (interleukin-6, IL-6), the microbial metabolite 5-(3′,4′-dihydroxyphenyl)-γ-valerolactone (M1), and *in vitro* differentiation experiments with human mesenchymal stromal cells (hMSCs).

**Results:**

Within the Pycnogenol^®^ group, serum CTX-1 levels were significantly reduced after both three (*p* = 0.0327) and 6 months (*p* = 0.0227) compared to baseline, while no between-group differences were observed at any visit. No significant changes were observed in P1NP concentrations. Additionally, a significant reduction from baseline was observed for NTX-1 serum levels in the Pycnogenol^®^ group at the end of the active-treatment extension (*p* = 0.0100). Salivary M1 concentrations were significantly higher following 3 months of Pycnogenol^®^ supplementation compared to placebo (*p* = 0.0289) and showed an inverse association with CTX-1 concentrations (*r* = −0.216, *p* = 0.013, *n* = 131).

**Conclusion:**

Dietary supplementation with Pycnogenol^®^ was associated with reductions in key bone resorption markers within the treatment group, while metabolite concentrations in saliva increased. Furthermore, Pycnogenol^®^ components enhanced the osteogenic differentiation of hMSCs *in vitro*. These findings suggest a potential protective effect of Pycnogenol^®^ on bone turnover in osteopenia and clinically stable osteoporosis.

**Clinical trial registration:**

https://drks.de/search/de/trial/DRKS00026630, DRKS00026630.

## 1 Introduction

Osteoporosis is a chronic metabolic bone disease with a progressive and above average decline in bone mineral density (BMD) and deterioration of bone trabecular microstructure, leading to an increased risk of fragility fractures. Fractures cause pain, reduce mobility and might lead to disability or premature death. Osteoporosis affects primarily the aging population with postmenopausal women being at particular risk. Recently, it has been estimated that 23 million women and men in the European Union are at high risk for osteoporotic fractures and that one-third of women and at least one-sixth of men will suffer such a fracture in their lifetime (1).

The operational definition of osteoporosis is based on BMD measurements using Dual X-ray Absorptiometry (DXA) at the lumbar spine and the proximal femur with individual results at least 2.5 standard deviations below reference data for 20–40 years old, healthy, sex-matched reference population (T-Score ≤ − 2.5). Bone mass between 1.0 to and 2.5 D below reference level is defined as osteopenia.

While the treatment of osteoporosis encompasses various pharmacological options to reduce fracture risk, patients with osteopenia or patients with BMD-defined osteoporosis but otherwise stable disease and fracture risk below guideline defined treatment thresholds are typically managed with non-pharmacological lifestyle modifications such as healthy dietary patterns, exercise and supplementation with vitamin D and calcium. This pre-therapeutic stage of incipient fracture risk due to osteoporosis may be considered a window of opportunity for a modification of the trajectory of BMD decline by positively influencing the remaining bone remodeling capacity (2).

In the context of attenuation of disease progression and preserving bone density, dietary supplements such as polyphenols have attracted considerable attention. The current knowledge regarding molecular compound bioactivity and evidence from clinical studies has been summarized in several recent reviews (2–5). When the impact of plant-derived polyphenol extracts on bone health is researched, it is essential that the extract constituents are known and standardized so that any observed bioactivity can be translated to other study populations.

A well-defined polyphenolic extract containing 65–75% oligomeric procyanidins is the French maritime pine bark extract Pycnogenol®. Details of extract identity, purity and content have been monographed as a dietary supplement in the United States Pharmacopeia (USP). Safety and effects have been investigated in randomized, double-blind placebo-controlled clinical studies (6) and the human pharmacokinetics has been explored (7). Interestingly, effects of Pycnogenol® can be partly attributed to the gut microbial metabolite 5-(3′,4′-dihydroxyphenyl)-γ-valerolactone (M1). Although the impact of a dietary supplementation with Pycnogenol® on the course of bone loss in osteopenia or clinically stable osteoporosis has not been examined in humans yet, animal experiments with rodents suggested a protective effect of Pycnogenol® on bone loss, inhibitory effects on bone resorption and osteoclast differentiation (8–10).

An intensively investigated cell type which additionally contributes to bone health are bone marrow mesenchymal stem or stromal cells (MSCs) which are progenitors of both osteoblasts and adipocytes (11). Dynamic and balanced osteogenesis and adipogenesis is prevalent under physiological conditions and accounts for bone remodeling and integrity. The disruption of this homeostasis with impaired osteogenic differentiation and increased adipogenesis contributes to the development and progression of osteoporosis (12). Greater fattening of the bone marrow has been found to predict bone loss in senior women (13) and the risk of fragility fractures has been particularly linked to saturated marrow lipids (14).

The differentiation of MSCs is regulated by various factors. Aging processes and inflammation, particularly the activation of NF-κB, have been shown to disrupt the balance between osteogenesis and adipogenesis with a shift towards adipogenic differentiation. There is pronounced interest in chemical compounds regulating osteogenic/adipogenic differentiation (12). Some plant-derived polyphenols have been found to enhance osteogenesis (15).

The purpose of the present study was to investigate the potential impact of a dietary intervention with Pycnogenol® on bone metabolic markers in patients with osteopenia or clinically stable osteoporosis who were not intended for treatment with bone-specific pharmacotherapy. According to the international consensus and based on recent statements by, e.g., the International Federation of Clinical Chemistry and Laboratory Medicine (IFCC), markers recommended for evaluating bone turnover are CTX-1 (C-terminal telopeptide of type I collagen) as a bone resorption marker and P1NP (procollagen type I N propeptide) as a bone formation marker (16). Since both markers allow monitoring short-term changes and are safe to analyze in blood, urine or saliva (17) we chose them as primary endpoints. For adherence control and potential correlation analysis with effect parameters, concentrations of the Pycnogenol® metabolite M1 were analyzed in saliva samples of the participants. Additionally, the effects of selected constituents or metabolites of Pycnogenol® on the osteogenic differentiation of human mesenchymal stromal cells (hMSCs) were determined.

## 2 Materials and methods

### 2.1 Study design and participants

The present study was a prospective, randomized, placebo-controlled, double-blind, single-site clinical investigation with a two-arm parallel-group design, registered in the DRKS database (DRKS00026630; September 14th, 2021). The trial consisted of a placebo-controlled phase of 3 months and a subsequent active-treatment extension phase of the same length, during which participants initially allocated to placebo were switched to Pycnogenol® (Figure 1). It was conducted in accordance with the Declaration of Helsinki, concerning medical research in humans and received approval from the Ethics Committee of the University of Würzburg (Project identification code 218/20). A total of 67 patients were recruited at the Osteology Department, Klinik König-Ludwig-Haus, University Würzburg and participated in the study between October 2021 and January 2024. All participants provided written consent before any study-related procedures.

**Figure 1:**
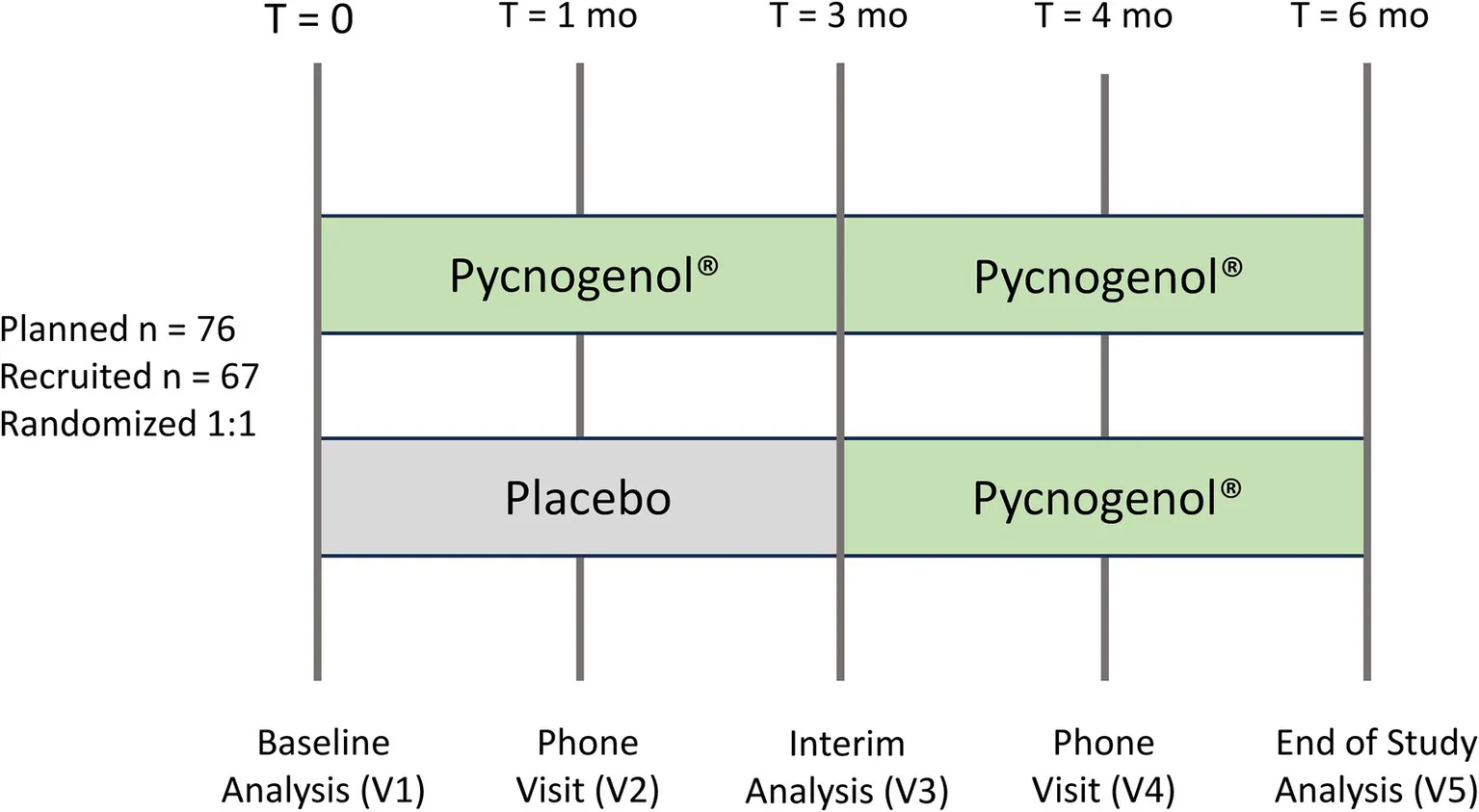
Study flow scheme illustrating the intervention course, time points, and types of visits.

### 2.2 Intervention and monitoring

The investigational product in this study was Pycnogenol®, a standardized extract of French maritime pine bark that complies with the quality standards of the United States Pharmacopeia for dietary supplements. Patients received either Pycnogenol® capsules (100 mg twice daily) or identically looking placebo capsules for 3 months. Participants were randomly assigned to one of the two groups in a 1:1 ratio. Randomization was performed using block randomization with a computer-generated list and blocks of 10 participants. Random numbers were generated with Excel and ranked. The patients were then assigned to either the Pycnogenol® or the placebo group according to the rank. Both the participants and the treating physicians (LS, FG, JM) were unaware of the group allocation, which ensured the double-blind design of the study. The randomization code was retained by the study evaluators (CS and PH). The study comprised five visits, three on-site visits (V1, V3 + V5) and two remote consultations (V2 + V4) (see study flow scheme, Figure 1). During the first in-person visit (V1) following provision of informed consent clinical and patient-related data were collected. After assessment for eligibility participants were randomized, and the assigned intervention started. Patients were also asked to complete two quality-of-life (SF-36v2 and QUALEFFO-41) and one nutrition questionnaire (18). At the next on-site visit (V3), 3 months after the start of the study, the clinical and laboratory parameters and questionnaires were reassessed. This visit also represented a prespecified interim analysis, which was based on the change in bone turnover markers of the first 40 participants having achieved that milestone. The analysis was carried out by the study evaluators (CS and PH), who had access to the randomization code, while participants and the physicians responsible for patient management (FG, JM, LS) remained blinded and the results were not disclosed to them. The analysis served exclusively to decide whether the trial should be continued and involved no formal hypothesis testing, so that no adjustment of the significance level was necessary. Since the change in bone turnover markers proved numerically more favorable under Pycnogenol® than under placebo, the trial was carried on unchanged with respect to sample size and final analysis. Following V3, those participants who were in the placebo group were switched to the active treatment arm for the second study phase (3 months). The final in-person visit (V5) was scheduled 6 months after treatment initiation and included follow-up assessments of clinical and laboratory data and questionnaires. In between the above-mentioned on-site visits, participants were contacted by phone at V2 and V4 at 6 weeks after V1 and V3 to check for adverse events and confirm adherence to study intervention.

The number of patients to be studied was calculated to achieve a statistical power of 80% and a significance level of 5%. Based on a standard deviation of SD = ± 10 μg/L for P1NP and SD = ± 0.25 μg/L for CTX-1 (19), which were the primary endpoint markers of the study, and clinically relevant changes of 20% corresponding to effect sizes of 0.7 and 0.8, the required sample size was determined to be 68 for P1NP and 52 for CTX-1, respectively. Considering a drop-out rate of 10%, and even sample size per treatment arm, a total of 76 participants was pursued. Evaluation of laboratory values at visits 1/3/5 was based on peripheral blood samples including assessment of hematology/blood count, liver-enzymes, kidney, electrolytes, alkaline phosphatase (ALP), parathyroid hormone 1–84 (PTH), intact fibroblast growth factor-23 (iFGF-23) and vitamin D. Additionally, at visits 1, 3 and 5, peripheral venous blood samples were drawn for specific biomarker analysis with relevance for bone metabolism. The investigational markers were analyzed either at the Institute of Pharmacy at the University of Würzburg or at the Orthopedic Clinic. Here, we report the results of various bone turnover markers and the saliva concentration of a specific Pycnogenol® metabolite (M1), analyzed at baseline, 3 and 6 months after the start of the intervention, as well as the outcomes of cell culture experiments. The additional bone turnover and inflammatory markers as well as the salivary concentration of the Pycnogenol® metabolite M1 were prespecified secondary endpoints, whereas the cell culture experiments and the correlation analyses were of exploratory nature.

### 2.3 Inclusion and exclusion criteria

Men and women, aged 18 years or older, with a 10-year fracture risk ≥ 20% according to the National German Guidelines for Diagnosis and Treatment of Osteoporosis for postmenopausal women and men ≥ 60 years of age and a DXA T-Score ≤ − 1.0 at either total hip, femoral neck or lumbar spine were eligible to participate in the study. Exclusion criteria were a current or ongoing treatment with any bisphosphonate, denosumab, strontium ranelate, teriparatide or romosozumab and an osteoporotic fracture within 3 months prior to enrollment. Participants were excluded if they had a present medical indication for treatment of their osteoporosis according to applicable national guidelines with the following exceptions: patient refuses to take such medication or patient cannot be treated due to contraindications or an unfavorable risk/benefit ratio or patient was treated previously with such medication but to avoid long-term treatment associated side-effects pausing of treatment is being considered. Also, patients were not eligible if they were taking nutritional supplements.

### 2.4 Bone turnover markers and metabolite levels

Serum samples were collected to quantify the primary endpoint markers CTX-1 and P1NP, as well as additional bone resorption markers such as N-terminal telopeptide of type I collagen (NTX-1) and tartrate-resistant acid phosphatase 5b (TRAP5b), the bone formation marker bone sialoprotein (BSP), and the inflammatory marker interleukin-6 (IL-6). In addition, the concentration of the specific Pycnogenol® metabolite M1 (5-(3,4-dihydroxyphenyl)-γ-valerolactone) was determined in saliva (20). All blood and saliva samples were obtained during the on-site visits between 9 a.m. and 1 p.m., and sampling times were kept consistent across visits. Participants were instructed to attend in a fasting state. Blood samples were collected using the BD Vacutainer® SST II Advance (Becton Dickinson GmbH, Heidelberg, Germany) and centrifuged at 2,000×*g* for 10 min at 4 °C to separate serum, which was aliquoted, immediately shock-frozen, and stored at −80 °C until analysis. Saliva was collected using the SalivaBio Oral Swab (Biozol Diagnostica GmbH, Eching, Germany) by placing the swab under the tongue and holding it there for approximately 5 min. After collection the swab was immediately stored at 4 °C, before being centrifuged at 4,000×*g* for 10 min at 4 °C. The collected saliva samples were then aliquoted, frozen in liquid nitrogen, and kept at −80 °C until further analysis. To quantify the concentrations of the bone turnover and the inflammatory biomarkers in serum, enzyme-linked immunosorbent assay (ELISA) kits were used. Human CTX-1, P1NP and bone sialoprotein kits were purchased from Immundiagnostik AG (Bensheim, Germany). Human NTX-1 (Abbkine), TRAP5b (Biorbyt) and IL-6 (Biorbyt) kits were all obtained from Biozol Diagnostica GmbH (Eching, Germany). Serum samples were analyzed according to the manufacturers’ protocols. A previously validated liquid chromatography tandem mass spectrometry (LC–MS/MS) method was used to assess the Pycnogenol® metabolite in saliva (20).

### 2.5 Cell culture experiments

#### 2.5.1 Chemicals and reagents

The Pycnogenol® compounds taxifolin, ferulic acid, caffeic acid, gallic acid, trans para-coumaric acid and 3,4-dihydroxybenzoic acid (protocatechuic acid) were all obtained from Merck KGaA (Darmstadt, Germany). The gut microbial metabolite M1 (5-(3,4-dihydroxyphenyl)-γ-valerolactone) was purchased from Cayman Chemical (Ann Arbor, USA). The Pycnogenol® constituents’ conjugates p-coumaric acid 4-O-sulfate, 3,4-dihydroxybenzoic acid 3-O-sulfate and ferulic acid 4-O-sulfate were bought from Biosynth Ltd. (Compton, UK). Caffeic acid 3-Beta-D-glucuronide was obtained from LGC Standards (Teddington, UK). Cell culture media Dulbecco’s Modified Eagle Medium/Nutrient Mixture F-12 (DMEM/F-12) and DMEM High Glucose were sourced from Capricorn Scientific GmbH (Ebsdorfergrund, Germany). Cell culture additives L-ascorbic acid 2-phosphate, ß-glycerophosphate, Dexamethasone and Penicillin–Streptomycin, Dulbecco’s phosphate-buffered saline (PBS), Trypsin–EDTA solution and DMSO were all obtained from Merck KGaA (Darmstadt, Germany). Human serum was acquired from the Bavarian Red Cross Blood Donor Service (München, Germany) and 10% fetal bovine serum from Anprotec (Bruckberg, Germany). Fixative solution paraformaldehyde 4% in PBS was purchased from Morphisto GmbH (Offenbach, Germany). An Alizarin Red S Staining Quantification Assay was sourced from ScienCell Research laboratories (Carlsbad, USA). Cell culture plates were obtained from Greiner Bio-One GmbH (Frickenhausen, Germany).

#### 2.5.2 Osteogenic differentiation of hMSCs and Alizarin Red staining

HMSCs (*n* = 3, a 64-year-old Caucasian woman and two Caucasian men aged 60 and 62) were derived from the femoral head bone marrow obtained from the Orthopaedic Hospital König-Ludwig-Haus under the Ethical Grant Number 186/18, Date 13.11.2018 approved by the Ethics Committee of the University of Würzburg. Cells were isolated by a standardized protocol (21, 22), expanded and used in this study at passage 1–3. Briefly, bone marrow preparations were washed with Dulbecco’s modified Eagle’s Medium/Nutrient Mixture F-12 (DMEM/F12, Thermo Fisher Scientific) supplemented with 10% FBS, 100 U/mL penicillin, 100 μg/mL streptomycin, and 50 μg/mL ascorbate, and centrifuged at 1200 rpm for 5 min. The pellet was reconstituted in medium for 10–20 s and washed two times, and the supernatants of the washing steps containing the released cells were collected. Cells were centrifuged and seeded at a density of 1.5 × 10^9^ cells per 175 cm^2^ culture flask. Adherent cells were washed after 2–4 days and cultivated until confluence in a humidified atmosphere with 5% CO_2_. Next, hMSCs were grown in the above described medium at standard conditions of 37 °C under 5% CO_2_ in a humidified cell culture. The culture medium was replaced twice a week. After reaching 80–90% confluence, the cells were harvested by trypsinization and seeded in 6-well plates at a concentration of 5 × 10^3^ cells/cm^2^ using the same culture medium. For osteogenic differentiation, hMSCs at passage 2 or 3 (P2 or P3) were maintained until they reached 70% confluence. Subsequently, cells were induced to differentiate into osteoblasts by changing the culture medium to osteogenic medium, which consisted of DMEM High Glucose, supplemented with 100 U/mL penicillin, 100 μg/mL streptomycin, 50 μg/mL L-ascorbic acid-2-phosphate, 10 mM ß-glycerophosphate, and 100 nM dexamethasone. Pooled human serum from three healthy donors, acquired from the Bavarian Red Cross Blood Donor Service, was used as the serum component. The cells were simultaneously treated with Pycnogenol® constituents or corresponding conjugates at final concentration of 2.0 μg/mL or with 1.0 μg/mL of M1 (further details are given in the Results section). The stock solutions of the investigational compounds were prepared in DMSO and stored at −80 °C. As DMSO was present in the culture dishes at a final concentration of 0.1%, differentiation medium with 0.1% DMSO served as control. Prior to the differentiation experiments, the nontoxicity of these concentrations of Pycnogenol® substances and DMSO was verified using a Neutral Red Assay based on the work of Repetto et al. (23). The osteogenic differentiation of hMSCs was carried out over a period of 21 days; the medium was renewed twice a week containing freshly added osteogenic stimuli, pooled human serum and plant extract solutions. On day 21 of osteogenic differentiation, the cells were assessed for calcium deposits using Alizarin Red staining. The staining and subsequent quantification were conducted employing an Alizarin Red S Staining Quantification Assay according to the manufacturer’s instructions (Provitro, Berlin, Germany). In brief, hMSCs were washed three times with PBS and then fixed in 4% paraformaldehyde/PBS for 15 min at room temperature. Following fixation, the cells were washed three times with distilled water and stained with 40 mM Alizarin Red solution for 20 min at room temperature. After the removal of the dye, cells were washed five times with distilled water. Finally, images of the cells were taken using an Axiovert 100 microscope (Carl Zeiss Microscopy GmbH, Jena, Germany). For Alizarin Red quantification, the stain was eluted with 10% acetic acid and then neutralized with 10% ammonium hydroxide. Absorbance was measured at 405 nm using an Infinite M Plex microplate reader (Tecan Group Ltd., Männedorf, Switzerland).

### 2.6 Statistical analysis

Data were analyzed using descriptive statistics. Typically, mean and standard deviation (SD) were calculated. All calculations were performed using the GraphPad® Prism software, version 11 for windows (GraphPad Software, Inc., La Jolla, CA, USA). Before statistical testing, the ROUT method implemented in GraphPad® Prism was employed to perform a two-tailed outlier test with a *Q* value of 1%, identifying and removing any outliers from the dataset. The outlier analysis was applied uniformly to all assessed parameters and independently of treatment allocation. The procedure was not prespecified in the study protocol. For comparison of the study participants´ basic demographic characteristics a *t*-test (for continuous variables) and a Fisher’s exact test (for categorical variables) was used.

Biomarker and metabolite concentrations were analyzed using a mixed-effects model (REML) fitted with treatment group, time and their interaction as fixed effects and participant as a random effect, thereby including all available observations without requiring complete data at every visit. Pairwise comparisons within and between groups were adjusted for multiplicity using Tukey’s method within each parameter; no correction was applied across different parameters.

Cell culture data were analyzed separately. In the first series of experiments, two conditions were compared using a paired *t*-test. In the second series, comparisons between more than two groups were performed by ordinary one-way ANOVA with Tukey’s *post hoc* correction for multiple comparisons.

The association between variables was evaluated using the Spearman’s rank correlation coefficient, pooling observations from all time points and both treatment arms; the number of analyzed pairs is reported for each correlation. Statistical significance was defined as *p*-values < 0.05. The study was powered for the primary endpoints CTX-1 and P1NP; the remaining outcomes are reported as prespecified secondary or exploratory endpoints without confirmatory claim.

## 3 Results

### 3.1 Participant flow and baseline characteristics

Following eligibility screening, a total of 67 patients were enrolled into the study and randomized to receive either 200 mg Pycnogenol® (*n* = 33) per day or placebo capsules (*n* = 34). The proportion of participants who discontinued their assigned medication during the study (dropouts) was balanced between the groups: 9.1% (*n* = 3) in the Pycnogenol® group and 8.8% (*n* = 3) in the placebo group. The participant flow is shown in Figure 2, aligned with the Consolidated Standards of Reporting Trials (CONSORT) guidelines. Two participants discontinued the study medication because of an adverse event possibly related to the study medication: one participant in the Pycnogenol® group reported dizziness and one participant in the placebo group experienced repeated vomiting. Both events were rated as mild. Two further participants per group were lost to follow-up without a reported reason. All participants in the study were women. The mean age (60.67 ± 13.12 years versus 63.09 ± 8.04 years) and BMI (24.78 ± 4.77 kg/m^2^ versus 24.19 ± 3.49 kg/m^2^) were comparable in both groups. There was no statistically significant difference between the groups in any other variable such as smoking behavior, alcohol consumption, vegetarian diet, bone mineral density, fractures within the last 10 years and diagnosis of an inflammatory disease (Table 1).

**Figure 2:**
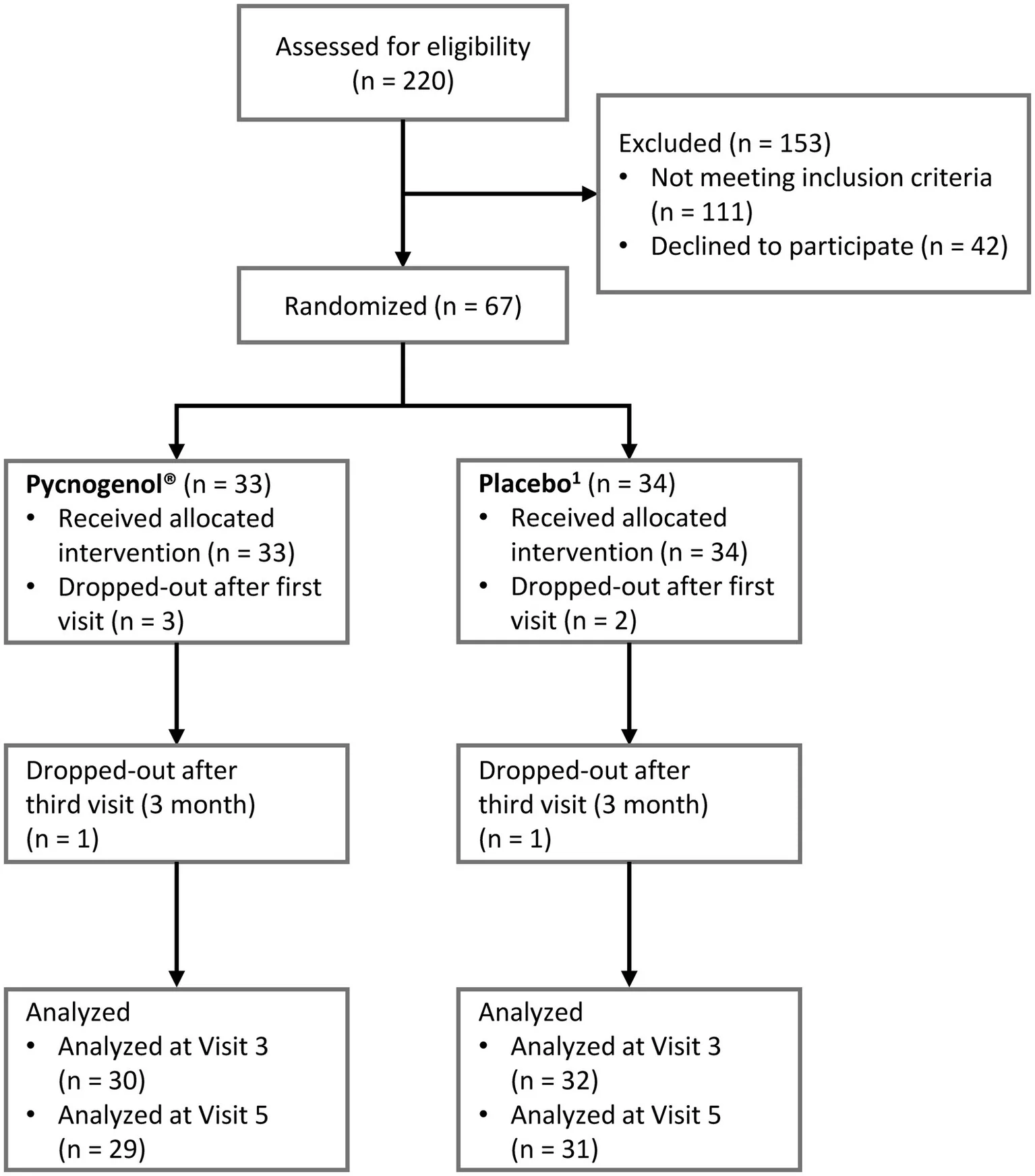
Consolidated Standards of Reporting Trials (CONSORT) flow chart illustrating the participant flow through the study, including enrollment, allocation to intervention groups (Pycnogenol^®^ and placebo), follow-up, and analysis. ^1^6-month value: switch to Pycnogenol^®^.

**Table 1:** Demographic and lifestyle characteristics of the study participants.

Variables	Pycnogenol^®^ group^1^ (*n* = 33)	Placebo group^1^ (*n* = 34)	*p*-value^2^
Age (years), mean ± SD	60.67 ± 13.12	63.09 ± 8.04	0.6115 (M)
BMI (kg/m^2^), mean ± SD	24.78 ± 4.77	24.19 ± 3.49	0.5967 (T)
L1-L4^3^ (T-Score), mean ± SD	−1.61 ± 0.95	−1.79 ± 1.12	0.5175 (T)
L femur (T-Score), mean ± SD	−1.41 ± 0.72	−1.70 ± 0.74	0.1326 (T)
Vegetarian diet, *n* (%)			
Yes	3 (9.1)	2 (5.9)	>0.9999 (F)
No	24 (72.7)	25 (73.5)	
Missing	6 (18.2)	7 (20.6)	
Smoking status, *n* (%)			
Yes	3 (9.1)	5 (14.7)	0.7049 (F)
No	24 (72.7)	23 (67.6)	
Missing	6 (18.2)	6 (17.6)	
Drinking status, *n* (%)			
Yes	21 (63.6)	20 (58.8)	>0.9999 (F)
No	6 (18.2)	7 (20.6)	
Missing	6 (18.2)	7 (20.6)	
Fractures ≤ 10 years ago, *n* (%)			
Yes	11 (33.3)	7 (20.6)	0.2623 (F)
No	18 (54.5)	24 (70.6)	
Missing	4 (12.1)	3 (8.8)	
Inflammatory disease, *n* (%)			
Yes	7 (21.2)	13 (38.2)	0.1772 (F)
No	22 (66.7)	18 (52.9)	
Missing	4 (12.1)	3 (8.8)	

^1^Differences in individual parameters are based on incomplete data sets ^2^Compared between groups; Mann–Whitney test (M), two-tailed *t*-test (T), Fisher’s exact test (F). ^3^L1-L4: lumbar vertebrae 1–4 (standard DXA region of interest for lumbar spine BMD) *Significant *p*-value for *α* < 0.05.

### 3.2 Primary endpoint markers

Serum concentrations of the primary endpoint markers are shown in Table 2 and Figure 3. The number of observations excluded by the ROUT procedure is reported for all analyzed parameters by treatment group and visit in Supplementary Table S1.

**Table 2:** Concentrations of primary endpoint markers, determined in the Pycnogenol^®^ and placebo group.

Parameter	Measurement Time	Pycnogenol^®^ Mean ± SD	*n* ^1^	Placebo^2^ Mean ± SD	*n* ^1^	*p*-value^a^	Mixed-effects model *p*-values^c^
CTX-1 [pg/mL]	Baseline (V1)	417.8 ± 133.8	28	421.7 ± 171.7	32	0.9211	Time: 0.0150* Group: 0.9154 Time × Group: 0.9317
	3 months (V3)	373.7 ± 134.5	29	379.5 ± 131.5	32	0.8660	
	6 months (V5)	380.9 ± 149.9	29	383.4 ± 130.5	31	0.9458	
Within-group comparisons (paired observations)		Pycnogenol^®^		Placebo2			
V1 vs. V3 (Means ± SDs) *p*-value^b^, *n*^3^		417.8 ± 133.8 vs. 367.0 ± 131.8 0.0327*, *n* = 28		421.7 ± 171.7 vs. 379.5 ± 131.5 0.2399, *n* = 32			
V1 vs. V5 (Means ± SDs) *p*-value^b^, *n*^3^		416.9 ± 136.3 vs. 359.8 ± 125.7 0.0227*, *n* = 27		419.4 ± 174.1 vs. 383.4 ± 130.5 0.4795, *n* = 31			
V3 vs. V5 (Means ± SDs) *p*-value^b^, *n*^3^		367.0 ± 131.8 vs. 365.4 ± 126.9 0.9961, *n* = 28		375.3 ± 131.5 vs. 383.4 ± 130.5 0.9367, *n* = 31			

Parameter	Measurement Time	Pycnogenol^®^ Mean ± SD	*n* ^1^	Placebo^2^ Mean ± SD	*n* ^1^	*p*-value^a^	Mixed-effects model *p*-values^c^
P1NP [ng/mL]	Baseline (V1)	57.55 ± 16.32	28	66.23 ± 24.94	32	0.1127	Time: 0.1432 Group: 0.2014 Time × Group: 0.9388
	3 months (V3)	59.17 ± 16.41	28	66.76 ± 21.59	32	0.1284	
	6 months (V5)	62.77 ± 23.41	28	67.68 ± 20.40	30	0.3995	
Within-group comparisons (paired observations)		Pycnogenol^®^		Placebo2			
V1 vs. V3 (Means ± SDs) *p*-value^b^, *n*^3^		57.55 ± 16.32 vs. 59.17 ± 16.41 0.8106, *n* = 28		66.23 ± 24.94 vs. 66.76 ± 21.59 0.9755, *n* = 32			
V1 vs. V5 (Means ± SDs) *p*-value^b^, *n*^3^		58.00 ± 16.46 vs. 60.76 ± 21.26 0.4807, *n* = 27		64.48 ± 24.00 vs. 67.68 ± 20.40 0.4069, *n* = 30			
V3 vs. V5 (Means ± SDs) *p*-value^b^, *n*^3^		59.07 ± 16.71 vs. 60.76 ± 21.26 0.8221, *n* = 27		64.24 ± 18.68 vs. 67.68 ± 20.40 0.1725, *n* = 30			

^1^Number of participants with a quantifiable value at the respective visit, after exclusion of outliers by the ROUT procedure (*Q* = 1%); n therefore varies between visits. ^2^6-month value: Switch to Pycnogenol®. ^3^Number of participants with quantifiable values at both time points of the respective comparison; the corresponding means may therefore differ from the values shown for that visit in the upper part of each block.

Values are observed means ± SD. All *p-*values derive from one mixed-effects model (REML) per parameter, with treatment group, time and their interaction as fixed effects and participant as a random effect, using all available observations. Model estimates are therefore not derivable from the descriptive values shown.

^a^Between-group comparison at the respective visit; ^b^Within-group comparison between the two time points indicated. All pairwise comparisons were corrected for multiplicity using Tukey’s method within each parameter. ^c^Overall *p-*values for the fixed effects of the model. From V3 onward all participants received Pycnogenol®, so between-group comparisons at V5 reflect duration of exposure rather than treatment allocation and are descriptive.

*Significant *p*-value for *α* < 0.05.

**Figure 3:**
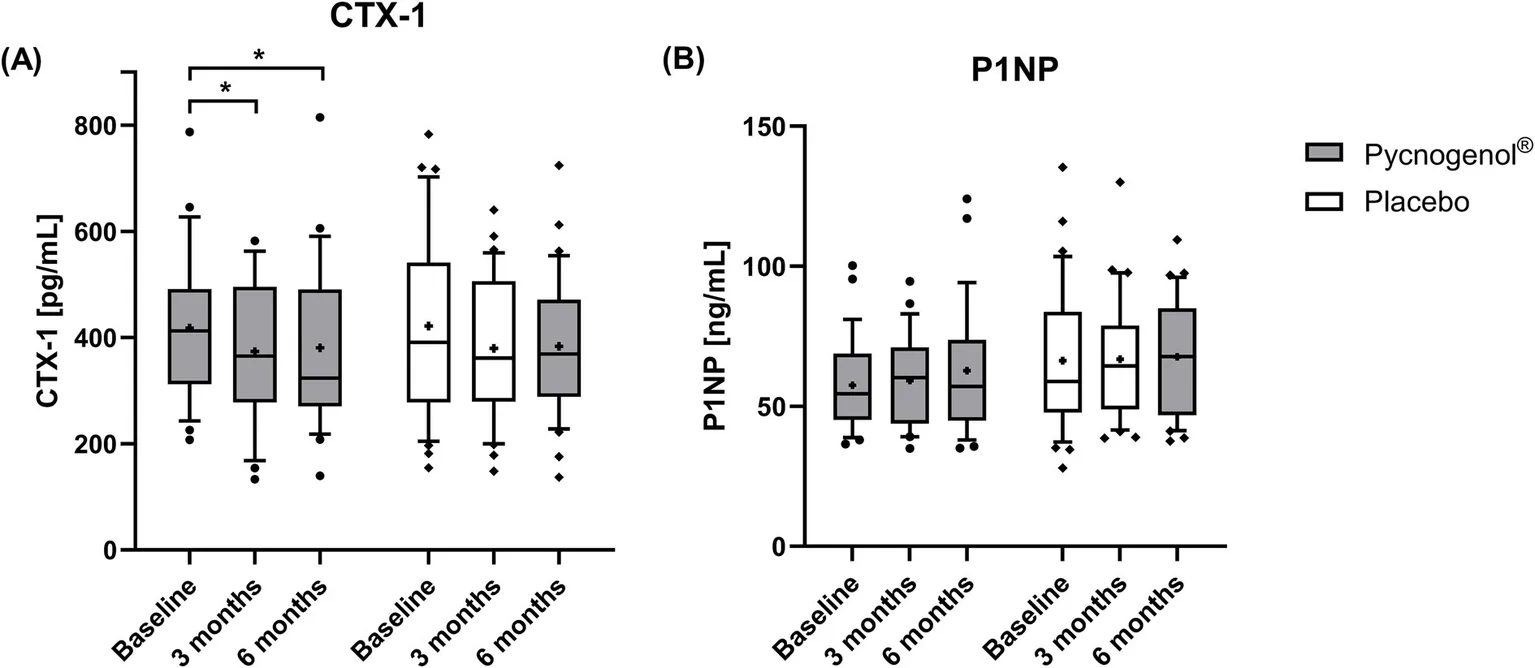
Primary endpoint markers (baseline, 3 and 6 months after start of intervention). Data are presented as Box-and-Whisker plot. Boxes show the interquartile range (Q1–Q3), with medians (line), means (+), whiskers (10th–90th percentiles), and outliers (circles/diamonds). Significant differences from baseline are indicated by asterisks (*), with a significance level of *α* < 0.05. Numbers of participants per visit are listed in Table 2. **(A)** C-terminal cross-linking telopeptide of type I collagen (CTX-1); **(B)** procollagen type 1 N propeptide (P1NP).

CTX-1 concentrations decreased over the study period in both study arms. In the Pycnogenol® group, they declined from 417.8 ± 133.8 pg/mL at baseline to 373.7 ± 134.5 pg/mL after 3 months and 380.9 ± 149.9 pg/mL after 6 months. In the group initially receiving placebo and switched to Pycnogenol® after 3 months, concentrations declined from 421.7 ± 171.7 pg/mL at baseline to 379.5 ± 131.5 pg/mL at the end of the placebo-controlled phase and were 383.4 ± 130.5 pg/mL after three further months of active treatment. The model indicated a significant effect of time (*p* = 0.0150), while no between-group differences were detected at any visit.

Within the Pycnogenol® group, the decrease from baseline reached statistical significance at the end of the placebo-controlled phase after 3 months (417.8 ± 133.8 pg/mL versus 367.0 ± 131.8 pg/mL; *p* = 0.0327) and after 6 months (416.9 ± 136.3 pg/mL versus 359.8 ± 125.7 pg/mL; *p* = 0.0227). The concentration remained essentially unchanged between 3 and 6 months (*p* = 0.9961). In the placebo group, the corresponding decreases did not reach statistical significance at any time point (*p* = 0.2399 and *p* = 0.4795) (Table 2; Figure 3A).

P1NP concentrations were numerically lower in the Pycnogenol® group at baseline (57.55 ± 16.32 ng/mL versus 66.23 ± 24.94 ng/mL, *p* = 0.1127) and remained essentially constant in both arms throughout the study, both after 3 months (59.17 ± 16.41 ng/mL versus 66.76 ± 21.59 ng/mL) and after 6 months (62.77 ± 23.41 ng/mL versus 67.68 ± 20.40 ng/mL). Neither within-group nor between-group comparisons revealed statistically significant differences at any visit (Table 2; Figure 3B).

### 3.3 Secondary endpoint markers

A trend resembling that of CTX-1 was observed for the other bone resorption marker NTX-1, for which the model likewise indicated a significant effect of time (*p* = 0.0013). In the Pycnogenol® group, the serum concentration decreased from 13.85 ± 6.25 nM bone collagen equivalent, BCE/L to 11.81 ± 6.41 nM BCE/L at the end of the placebo-controlled phase and showed a significant decline (*p* = 0.0100) to 9.40 ± 4.83 nM BCE/L after 6 months of supplementation. In the other study arm, the concentration remained essentially unchanged during placebo intake (12.98 ± 6.29 nM BCE/L versus 12.54 ± 5.85 nM BCE/L) and showed a slight decrease after the switch to Pycnogenol® (12.54 ± 5.85 nM BCE/L versus 11.14 ± 6.07 nM BCE/L), without reaching statistical significance. No difference between the groups were detected at any visit (Table 3; Figure 4A).

**Table 3:** Concentrations of secondary endpoint markers in serum, determined in the Pycnogenol^®^ and placebo group.

Parameter	Measurement Time	Pycnogenol^®^ Mean ± SD	*n* ^1^	Placebo^2^ Mean ± SD	*n* ^1^	*p*-value^b^	Mixed-effects model *p*-values^c^
NTX-1 [nM BCE/L]	Baseline (V1)	13.85 ± 6.25	30	12.98 ± 6.29	32	0.5861	Time: 0.0013* Group: 0.7299 Time × Group: 0.3720
	3 months (V3)	11.81 ± 6.41	30	12.54 ± 5.85	32	0.6402	
	*p*-value^a^ vs. V1	0.2640		0.9014			
	6 months (V5)	9.40 ± 4.83	29	11.14 ± 6.07	31	0.2235	
	*p*-value^a^ vs. V1	0.0100*		0.2317			
	*p*-value^a^ vs. V3	0.1776		0.2830			
TRAP5b [mIU/mL]	Baseline (V1)	4.01 ± 3.10	30	3.39 ± 2.24	30	0.3828	Time: 0.3403 Group: 0.7701 Time × Group: 0.2766
	3 months (V3)	4.01 ± 3.18	30	3.92 ± 3.00	30	0.9172	
	*p*-value^a^ vs. V1	>0.9999		0.2938			
	6 months (V5)	3.54 ± 2.44	29	3.55 ± 2.48	29	0.9898	
	*p*-value^a^ vs. V1	0.4800		0.6315			
	*p*-value^a^ vs. V3	0.5764		0.9227			
BSP [ng/mL]	Baseline (V1)	1.43 ± 0.87	27	1.52 ± 0.77	27	0.6890	Time: 0.3285 Group: 0.7928 Time × Group: 0.7937
	3 months (V3)	1.19 ± 0.41	26	1.41 ± 0.76	26	0.2059	
	*p*-value^a^ vs. V1	0.7986		0.3790			
	6 months (V5)	1.47 ± 0.84	26	1.47 ± 0.75	27	0.9937	
	*p*-value^a^ vs. V1	>0.9999		0.6364			
	*p*-value^a^ vs. V3	0.5116		0.9893			
IL-6 [ng/L]	Baseline (V1)	74.79 ± 60.25	27	59.66 ± 36.06	26	0.2715	Time: 0.2130 Group: 0.4856 Time × Group: 0.2503
	3 months (V3)	54.71 ± 45.64	24	57.28 ± 32.29	26	0.8208	
	*p*-value^a^ vs. V1	0.5542		0.8354			
	6 months (V5)	42.81 ± 20.50	21	57.67 ± 32.83	24	0.0728	
	*p*-value^a^ vs. V1	0.1392		0.9922			
	*p*-value^a^ vs. V3	0.7147		0.7383			
M1 [ng/mL]	Baseline (V1)	1.26 ± 1.31	22	0.95 ± 0.86	23	0.3595	Time: 0.0125* Group: 0.7493 Time × Group: 0.0009*
	3 months (V3)	2.30 ± 2.35	20	0.97 ± 1.13	22	0.0289*	
	*p*-value^a^ vs. V1	0.0735		0.3178			
	6 months (V5)	1.26 ± 1.16	21	2.56 ± 2.66	23	0.0407*	
	*p*-value^a^ vs. V1	0.8850		0.0090*			
	*p*-value^a^ vs. V3	0.1844		0.0478*			

^1^Number of participants with a quantifiable value at the respective visit, after exclusion of outliers by the ROUT procedure (Q = 1%); *n* therefore varies between visits. ^2^6-month value: Switch to Pycnogenol®.

Values are observed means ± SD. All *p-*values derive from one mixed-effects model (REML) per parameter, with treatment group, time and their interaction as fixed effects and participant as a random effect, using all available observations. Model estimates are therefore not derivable from the descriptive values shown.

^a^Within-group comparisons versus V1 or V3; ^b^between-group comparison at that visit. All pairwise comparisons were corrected for multiplicity using Tukey’s method. ^c^Overall *p-*values for the fixed effects of the model. From V3 onward all participants received Pycnogenol®, so between-group comparisons at V5 reflect duration of exposure rather than treatment allocation and are descriptive.

*Significant *p*-value for *α* < 0.05.

**Figure 4:**
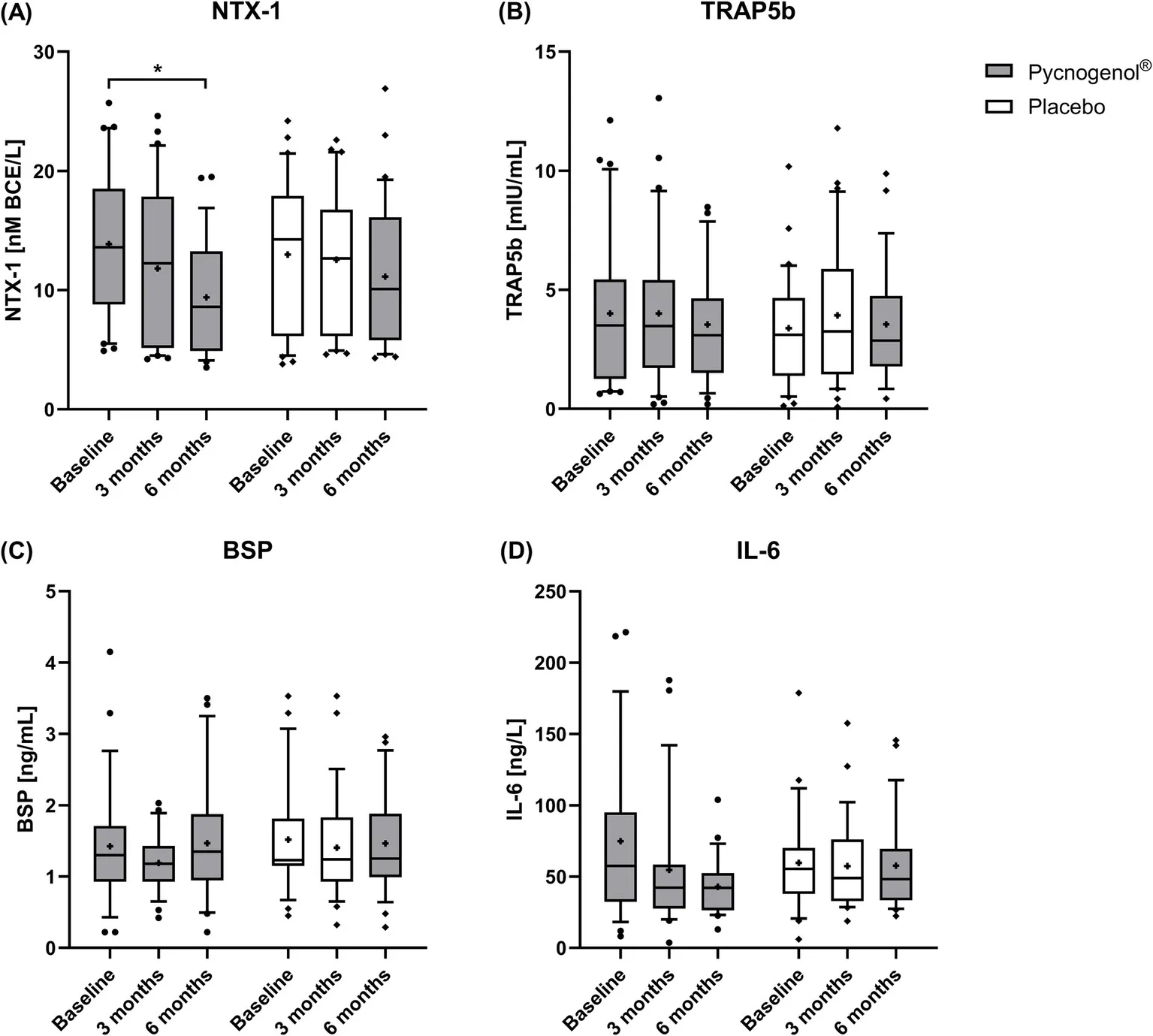
Secondary endpoint markers (baseline, 3 and 6 months after start of intervention). Data are presented as Box-and-whisker plot. Boxes show the interquartile range (Q1–Q3), with medians (line), means (+), whiskers (10th-90th percentiles), and outliers (circles/diamonds). Significant differences from baseline are indicated by asterisks (*), with a significance level of *α* < 0.05. Numbers of participants per visit are listed in Table 3. **(A)** N-terminal telopeptide of type I collagen (NTX-1); **(B)** tartrate-resistant acid phosphatase 5b (TRAP5b); **(C)** bone sialoprotein (BSP); **(D)** interleukin-6 (IL-6).

The serum TRAP5b concentration showed a slight, non-significant decrease when the plant extract was taken for a period of 6 months (4.01 ± 3.10 mIU/mL versus 3.54 ± 2.44 mIU/mL). In the placebo group, serum levels increased slightly from 3.39 ± 2.24 mIU/mL to 3.92 ± 3.00 mIU/mL and returned to 3.55 ± 2.48 mIU/mL following the switch to Pycnogenol®. None of the comparisons reached statistical significance (Table 3; Figure 4B).

Bone sialoprotein remained almost constant throughout the study in both arms. In the Pycnogenol® group the bone sialoprotein level was 1.43 ± 0.87 ng/mL at baseline, 1.19 ± 0.41 ng/mL at 3 months, and 1.47 ± 0.84 ng/mL after 6 months of Pycnogenol® administration. In the group initially taking placebo, bone sialoprotein concentrations were 1.52 ± 0.77 ng/mL at baseline, 1.41 ± 0.76 ng/mL at 3 months and 1.47 ± 0.75 ng/mL after the switch to the plant extract (Table 3; Figure 4C).

The inflammation-related biomarker IL-6 showed a marked numerical decrease in the Pycnogenol® group over the study period, from 74.79 ± 60.25 ng/L at baseline to 54.71 ± 45.64 ng/L at the end of the placebo-controlled phase and 42.81 ± 20.50 ng/L after 6 months, corresponding to a reduction of approximately 40%. However, the decrease did not reach statistical significance (*p* = 0.1392). IL-6 levels in the other study arm remained virtually unchanged over the entire study period, from 59.66 ± 36.06 ng/L at baseline to 57.28 ± 32.29 ng/L after 3 months of placebo and to 57.67 ± 32.83 ng/L after additional 3 months of Pycnogenol® (Table 3; Figure 4D).

### 3.4 Salivary metabolite concentrations

In addition to bone turnover markers, metabolite concentrations in saliva were also determined. However, not all participants provided sufficient saliva volumes for analysis. The metabolite was determined in 69.7% of the participants in the Pycnogenol® group and 76.5% of the placebo group. The model indicated a significant effect of time (*p* = 0.0125) and a significant treatment-by-time interaction (*p* = 0.0009), reflecting divergent trajectories of M1 concentrations in the two study arms. At the end of the placebo-controlled phase after 3 months, M1 concentrations were significantly higher in the Pycnogenol® group compared to the placebo group (2.30 ± 2.35 ng/mL versus 0.97 ± 1.13 ng/mL, *p* = 0.0289). Moreover, patients who switched from placebo to Pycnogenol® for the subsequent 3 months experienced a significant elevation in M1 levels compared to baseline in the active-treatment extension (2.56 ± 2.66 ng/mL versus 0.95 ± 0.86 ng/mL, *p* = 0.0090). This increased M1 concentrations were even significantly higher than the 6-month levels in the Pycnogenol® group (2.56 ± 2.66 ng/mL versus 1.26 ± 1.16 ng/mL, *p* = 0.0407). In the Pycnogenol® group, mean salivary M1 concentrations almost doubled after 3 months in the placebo-controlled phase (1.26 ± 1.31 ng/mL versus 2.30 ± 2.35 ng/mL), but decreased at the final assessment (Table 3; Figure 5).

**Figure 5:**
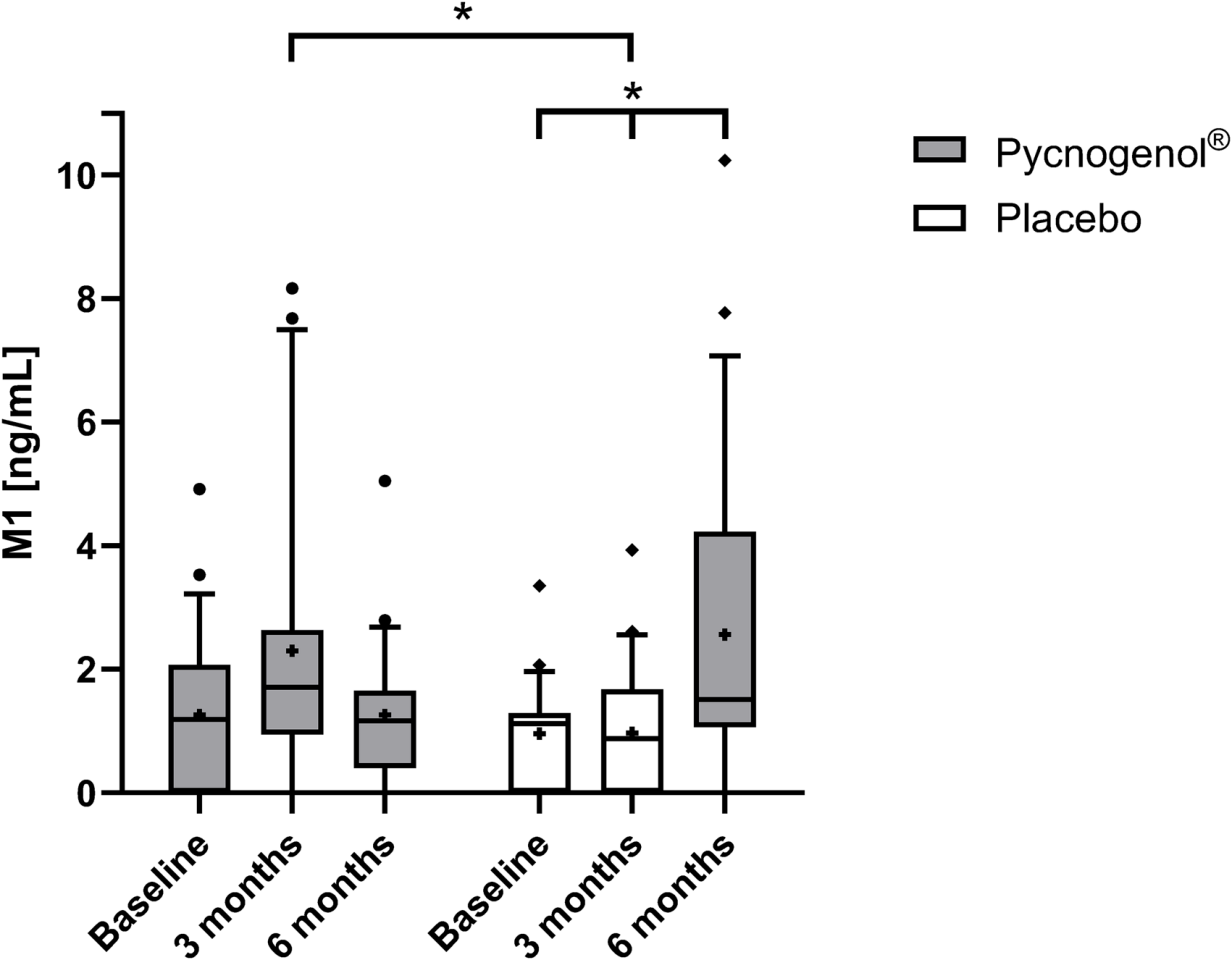
Salivary concentration of M1 in the Pycnogenol^®^ and placebo group (baseline, 3 and 6 months after start of intervention). Data are presented as Box-and-Whisker plot. Boxes show the interquartile range (Q1–Q3), with medians (line), means (+), whiskers (10th–90th percentiles), and outliers (circles/diamonds). Asterisks (*) indicate statistical significance at *α* < 0.05 for the comparison marked by the respective bracket. Numbers of participants per visit are listed in Table 3.

### 3.5 Correlation analysis

Bivariate correlation analysis using Spearman’s rank coefficient revealed statistically significant associations between bone turnover markers, IL-6, and the salivary metabolite M1 (Table 4). The primary endpoint marker CTX-1 showed a significant positive correlation with NTX-1 (*r* = 0.370, *p* < 0.0001, *n* = 180), while a significant negative correlation was observed with salivary M1 (*r* = −0.216, *p* = 0.013, *n* = 131). M1 concentrations in saliva further exhibited a weak but significant negative correlation with NTX-1 (*r* = −0.192, *p* = 0.028, *n* = 131), which in turn showed a significant positive correlation with the inflammation-related IL-6 (*r* = 0.307, *p* < 0.001, *n* = 147).

**Table 4:** Spearman’s rank correlation coefficients (*r*) and *p*-values for the associations between the serum bone turnover markers, inflammation-related IL-6, and salivary metabolite levels.

Variable 1	Variable 2	*n* ^1^	Spearman‘s *r*	*p*-value*
NTX-1	CTX-1	180	0.370	<0.0001
NTX-1	IL-6	147	0.307	<0.001
M1	CTX-1	131	−0.216	0.013
M1	NTX-1	131	−0.192	0.028

^1^Number of analyzed pairs for each correlation. *Significant *p*-value for *α* < 0.05.

### 3.6 Osteogenic differentiation of hMSCs and Alizarin Red staining

HMSCs were successfully differentiated and representative macroscopic Alizarin Red staining images, as well as microscopic images of stained and unstained cells, from stimulated and unstimulated, control cells are shown in Figure 6.

**Figure 6:**
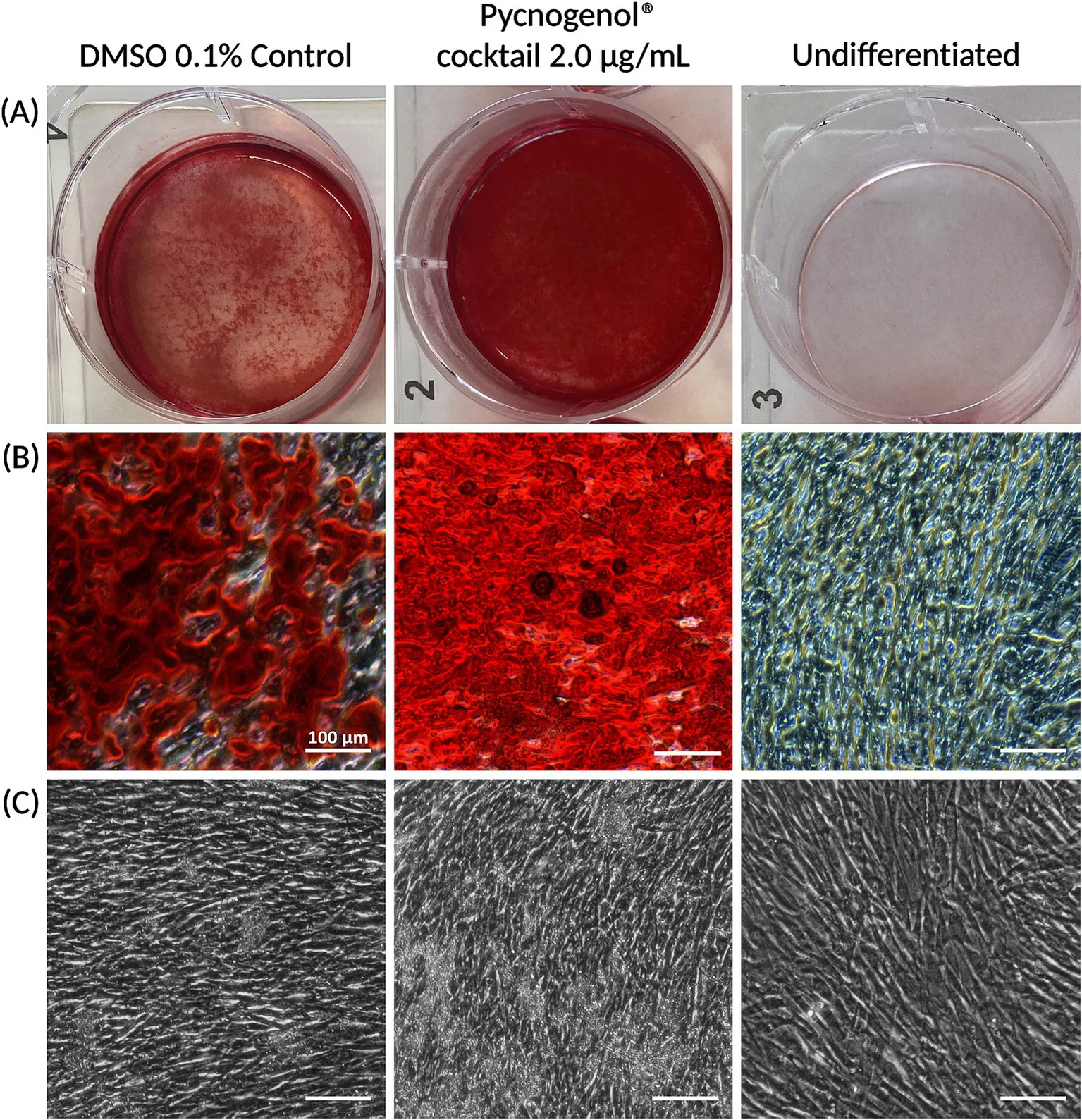
Representative Alizarin Red staining for calcium deposition in hMSCs. **(A)** Macroscopic images of stained 6-wells. **(B)** Microscopic bright field with phase-contrast images of Alizarin Red-stained hMSCS at day 21 of differentiation. Scale bar = 100 μm. **(C)** Microscopic phase-contrast images of hMSCs prior to Alizarin Red staining (day 21 of differentiation). Scale bar = 100 μm.

In the first experimental series, a cocktail of seven Pycnogenol® components was tested to assess its influence on the osteogenic differentiation of hMSCs, using DMSO 0.1% as vehicle control. For this purpose, the phenolic acids trans para-coumaric acid, 3,4-dihydroxybenzoic acid, ferulic acid, caffeic acid and gallic acid, the flavonoid taxifolin, and the gut microbiota-derived bioactive metabolite M1 were each added at a concentration of 2.0 μg/mL to the cell cultures. Three independent osteogenic differentiations were performed with hMSCs from the same donor (*n* = 1) (Figure 7) and demonstrated that the addition of the Pycnogenol® cocktail components elicited a statistically significantly higher mean Alizarin Red concentration compared to the control (2.627 vs. 1.0, *p* = 0.0449) and thus an increase in mineralization.

**Figure 7:**
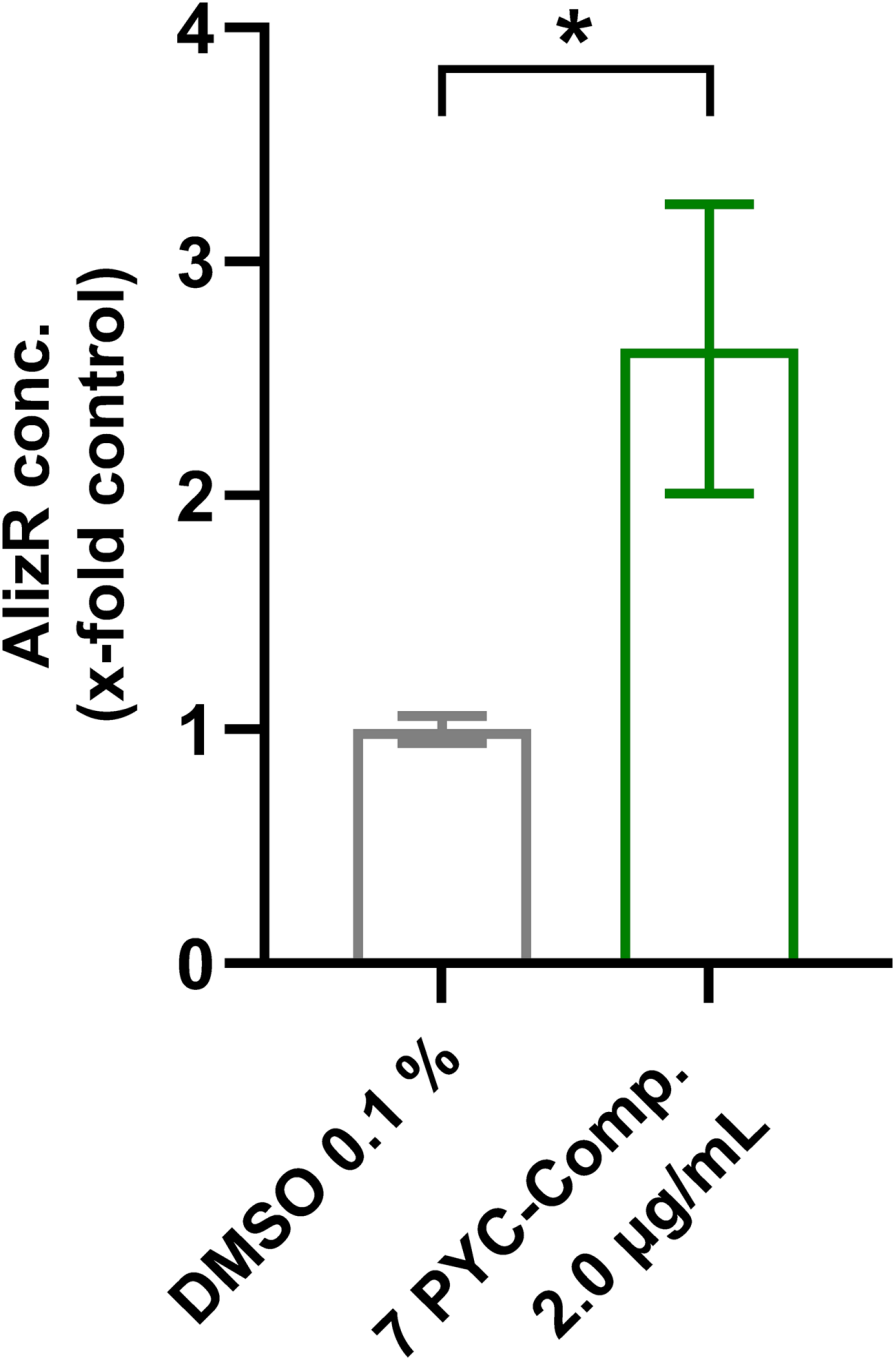
Osteogenic differentiation of hMSCs. Seven Pycnogenol^®^ components vs. DMSO control, summary of three independent osteogenic differentiations of one donor (*n* = 1). Data are presented as mean with standard deviations. Significant differences are indicated by asterisks (*), with a significance level of *α* < 0.05.

In a second series of experiments, the four Pycnogenol® constituents trans para-coumaric acid, 3,4-dihydroxybenzoic acid, ferulic acid and caffeic acid, as well as the four matching phase II conjugates para-coumaric acid 4-O-sulfate, 3,4-dihydroxybenzoic acid 3-O-sulfate, ferulic acid 4-O-sulfate and caffeic acid 3-beta-D-glucuronide, were investigated at a concentration of 2.0 μg/mL. In addition, the bioactive gut microbial metabolite M1 was used as single compound at a concentration of 1.0 μg/mL. Each of the experimental conditions resulted in higher Alizarin Red concentrations compared to the DMSO control. However, there was notable variability in mineralization both within donors (two independent differentiations for donors #1 and #3) and between donors (donor #1-3) (only a single differentiation performed for donor #2); each treatment was assessed in 3–5 replicates (*r* = 3–5 wells). Donor analysis of hMSCs revealed that for donor #3, the Pycnogenol® compounds and their corresponding conjugates exerted the greatest influence on osteogenic differentiation, leading to the highest mean Alizarin Red concentrations compared to control, whereas in hMSCs from donor #1 this effect was more pronounced for the individual substance M1. Donor #2 proved to be a weak differentiator, and the investigated compounds had no effect (Figure 8A). Having been identified as the most responsive donor, the two single osteogenic differentiations with hMSCs from donor #3 are shown in Figures 8B,C. Both the cocktail of four Pycnogenol® components at a concentration of 2.0 μg/mL as well as their four corresponding conjugates led to significantly higher Alizarin Red concentrations compared to the DMSO control (0.444 ± 0.202 mM and 0.441 ± 0.094 mM vs. 0.179 ± 0.034 mM, *p* = 0.0017) (Figure 8B). The single compound M1 at a concentration of 1.0 μg/mL was also superior to the control, although the difference was not statistically significant. In the second independent differentiation of the same donor, overall Alizarin Red concentrations were higher and showed consistent trends (Figure 8C), indicating a strong tendency for Pycnogenol® components and their corresponding phase II conjugates to enhance hMSC mineralization.

**Figure 8:**
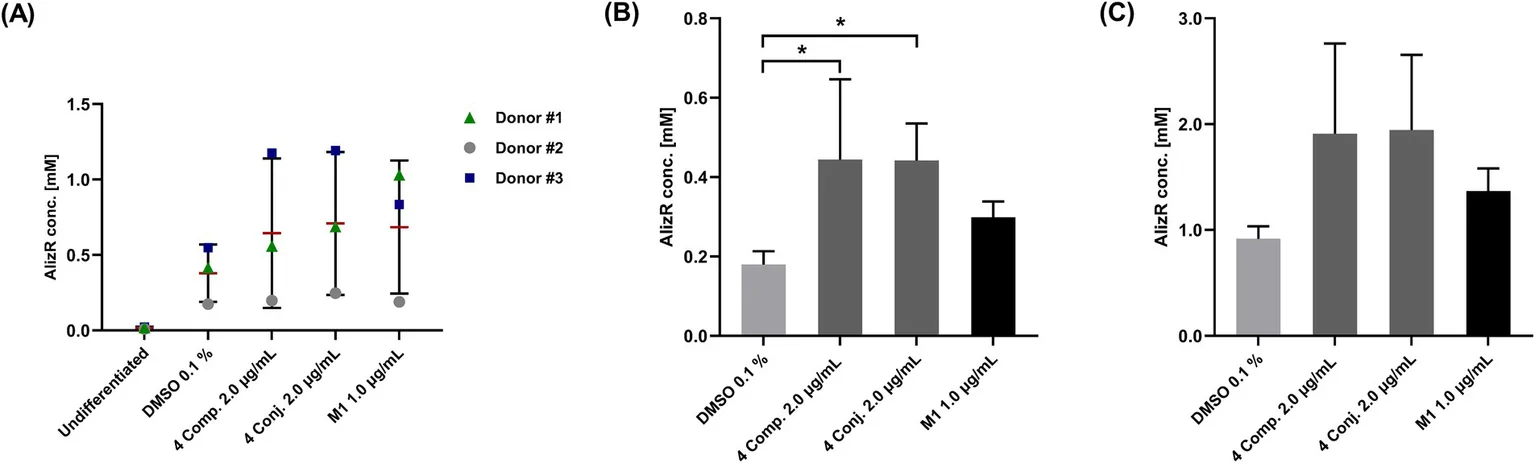
Osteogenic differentiation of hMSCs. **(A)** Four Pycnogenol^®^ phenolic acids, four matching phase II conjugates and M1 vs. DMSO control. Green triangles, grey circles and blue squares denote the mean concentrations for the respective hMSCs donors (*n* = 3). Red horizontal lines represent the average concentration across all donors, while whiskers show standard deviations. **(B)** First single osteogenic differentiation of donor #3 (*n* = 1) with four Pycnogenol^®^ phenolic acids, four matching phase II conjugates and M1 vs. DMSO control. Data are presented as mean with standard deviations of 5 wells (*r* = 5 wells). Significant differences are indicated by asterisks (*), with a significance level of *α* < 0.05. **(C)** Second single osteogenic differentiation of donor #3 (*n* = 1) with four Pycnogenol^®^ phenolic acids, four matching phase II conjugates and M1 vs. DMSO control. Data are presented as mean with standard deviation of 3–4 wells (*r* = 3–4 wells).

## 4 Discussion

The present study was the first to investigate the impact of dietary supplementation with procyanidin-rich French maritime pine bark extract (Pycnogenol®) on serum bone turnover markers and the presence of a specific Pycnogenol® metabolite in saliva in a randomized, double-blind trial comprising a 3-month placebo-controlled phase and a 3-month active-treatment extension, involving 67 women with low bone mineral density. The study design stipulated that the group originally assigned to placebo would switch to Pycnogenol® after the placebo phase to receive the dietary supplement for an additional 3 months. This approach was deliberately chosen to increase participants’ motivation for study participation and get additional information on reproducibility of a putative effect.

The standardized French maritime pine bark extract Pycnogenol® has shown modulatory effects on inflammatory pathways *in vitro* and *in vivo* with inhibition of NF-κB activation (24), decreased activity of cyclo-oxygenase (COX) enzymes and downregulation of cartilage-degrading mediators, such as matrix metalloproteinases (MMPs) in synovial fluid and cartilage (6, 25, 26). Bayer et al. recently demonstrated that Pycnogenol® intake significantly lowered salivary MMP-8 and serum IL-6 levels during non-surgical periodontal therapy, reflecting reduced gingival inflammation and alveolar bone resorption via inhibition of osteoclastogenesis and inflammatory cytokine activity (27).

The results of the present study revealed that supplementation with Pycnogenol® was accompanied by a significant decrease in key bone resorption biomarkers within the treatment group. CTX-1, a well-established marker of type 1 collagen degradation (28), was significantly reduced in the Pycnogenol® group compared to baseline, both at the end of the placebo-controlled phase and after 6 months. Notably, the absolute CTX-1 concentrations observed, both at baseline and after intervention, remained within the reference intervals for healthy women (e.g., 0.05–0.67 ng/mL for premenopausal; 0.09–1.05 ng/mL for postmenopausal) (29). This reduction suggests a decline in bone resorption activity and a favorable shift in bone turnover balance, potentially reflecting a protective effect on bone metabolism (30). The finding of a decrease in serum CTX-1, following administration of Pycnogenol® is further supported by a concordant, statistically significant reduction in serum NTX-1 levels after 6 months in the active-treatment extension. The results are in line with previous *in vivo* studies in which polyphenol-containing preparations led to a reduction in plasma CTX-1 levels (31–33). However, while our study demonstrated a significant decrease after only 3 months, it has to be acknowledged that there was also a non-significant decrease in the placebo-group, and that the between-group comparisons did not indicate a difference between the arms at any visit. Based on the data, the within-group reductions therefore do not yet provide sufficient evidence of a specific action of the intervention, and a larger trial would be required to confirm an effect of Pycnogenol® on bone resorption.

Coincident findings of unaltered TRAP5b levels further implicate that the observed decreases did not result from a reduction in the number of vital osteoclasts but rather their resorptive activity. In line with that, there is no reason to assume a negative effect in terms of bone’s remodelling capacity under such conditions. Accordingly, serum concentrations of the bone formation markers bone sialoprotein and P1NP reflecting bone turnover activity and osteoblastic bone formation, respectively, remained virtually constant in both groups throughout the study. This finding supports the concept that bone resorption as indicated by CTX-1 and NTX-1 might be mitigated in a favorable way regarding preservation of bone mass with Pycnogenol® while essential biological mechanisms of bone integrity remain intact. Studies with larger cohorts and longer intervention periods would nevertheless be required to detect potential effects on bone formation, which responds more slowly than markers of resorption (34, 35).

Earlier studies have identified serum IL-6 as a predictor of osteoporosis in postmenopausal women (36, 37) and experimental preclinical data, including IL-6 knockout and neutralization experiments in ovariectomized models, provide clear evidence for its direct involvement in osteoporosis pathogenesis (38, 39). Consistent with these findings, a significant positive correlation between IL-6 and NTX-1 was observed in the present study (*r* = 0.307; *p* < 0.001, *n* = 147), lending further support to the link between inflammatory activity and bone resorption. In the Pycnogenol® group, IL-6 concentrations declined by approximately 40% over the study period, whereas they remained unchanged in the other arm. However, the decrease did not reach statistical significance, which may be explained by the pronounced inter-individual variability reported for this parameter (40). The observation is nevertheless consistent with the anti-inflammatory properties described for Pycnogenol® (6) and would be compatible with a link between inflammatory activity and bone resorption, but requires confirmation in a study adequately powered for inflammatory endpoints.

Saliva analysis revealed that 3 months of Pycnogenol® supplementation increased the concentrations of the gut microbiota-derived metabolite M1 (5-(3,4-dihydroxyphenyl)-γ-valerolactone) both in the Pycnogenol® group as well as in the control group after switching to Pycnogenol®, which was reflected in a significant treatment-by-time interaction. Notably, M1 concentrations were significantly higher in the Pycnogenol® group than under placebo at the end of the placebo-controlled phase. Since M1 is formed exclusively by gut microbial degradation of procyanidins in the large intestine and reaches saliva from systemic circulation (7), salivary concentrations reflect systemic exposure rather than local formation, and their detection has been shown to indicate intake of the extract (27). Unexpectedly, M1 concentrations decreased again in the Pycnogenol® group after 6 months. Accordingly, a declining adherence over time is one possible explanation, although no adherence data were available to substantiate this assumption. The fact that despite decreased M1 concentrations after 6 months still improvements in clinical markers (CTX-1, NTX-1 and IL-6) were observed, indicates that additional bioactive metabolites or constituents add to the effects of Pycnogenol®. Nevertheless, the inverse associations between salivary M1 and the bone resorption markers CTX-1 and NTX-1would be consistent with a contribution of M1 to the effects of Pycnogenol®, although these correlation analyses were exploratory and do not permit conclusions on causality.

Current models of osteoporosis pathophysiology integrate factors such as oxidative stress, inflammation, hormonal imbalances and cellular senescence as interacting contributors to skeletal deterioration (41). In osteoporotic patients, an altered redox status with increased oxidative stress has been reported (42). Studies employing *in vitro*, animal, and human models have found that the polyphenol-rich pine bark extract has pronounced antioxidant activities (43, 44) and systemic anti-inflammatory effects (6). Pycnogenol® may additionally benefit bone health by modulating the “gut-bone-axis,” via effects on gut microbiota composition, microbial metabolites and gut-derived immune or endocrine signaling (45).

An impaired osteogenic differentiation of bone marrow MSCs at the expense of an increased adipogenesis has been suggested to contribute to the development and progression of osteoporosis (12), and plant-derived polyphenols might play a role in balancing osteogenic/adipogenic differentiation (15). Therefore, the effects of selected constituents and metabolites of Pycnogenol® on the osteogenic differentiation of human MSCs were determined in the present study. In the course of these experiments, experimental series with a single compound, compound mixtures and matching phase II metabolites, such as sulfate and glucuronic acid conjugates, which are typically present *in vivo* (7) were performed. Three independent differentiation experiments with hMSCs from the same donor using a mixture of seven Pycnogenol® components revealed a significantly enhanced osteogenic differentiation of hMSCs compared to control. In the subsequent experiments using cells from three different donors, considerable donor variability was observed. These results highlight the challenges posed by biological variability, in particular in donors with advanced age (60–70 years) which can influence hMSC differentiation capacity (46, 47). At the same time, the donor age corresponded closely to that of the population studied in the clinical part of the trial, so that the experimental setting reflected the intended translational context. Since the osteogenic capacity of bone marrow-derived MSCs declines with donor age (47), the responses observed in the present investigation may underestimate those in younger donors, which also limits the transferability of these findings to younger populations. Hence, further experiments with younger donors are warranted. Moreover, our findings suggest that responsive donors should first be identified. In both osteogenic differentiations using hMSCs from the best responder, four individual Pycnogenol® constituents and their corresponding phase II conjugates enhanced osteogenic differentiation. These results are consistent with the previous observation that serum of volunteers who ingested Pycnogenol® significantly diminished the activation of NF-kB (24) which plays a major role in disruption of osteogenesis (12). This mechanism is supported by a report of ferulic acid, a bioactive constituent of Pycnogenol®, promoting osteogenic differentiation of human periodontal ligament stem cells (hPDLSCs) by inhibiting the NF-kB signaling pathway (48). Future mechanistic studies with highly responsive donors would be desirable to investigate in detail the observed effects at the cellular and molecular levels.

Our findings are in line with previous research on *in vitro* experiments with polyphenolic compounds (49, 50). For instance, Lin et al. (51) demonstrated that (−)-epigallocatechin-3-gallate, the predominant catechin in green tea, enhanced osteogenic differentiation of human bone marrow mesenchymal stem cells, while it was shown that M1 activates Sirtuin 1 (SIRT1)-mediated autophagy to attenuate H_2_O_2_-induced inhibition of osteoblast differentiation in MC3T3-E1 cells (52). However, to the best of our knowledge, the present study investigated for the first time phase II conjugates of polyphenols (predominantly sulfates) along with their parent compounds for their influence on the osteogenic differentiation of hMSCs. After intake of Pycnogenol®, a substantial proportion of the extract’s constituents and their metabolites were found as phase II conjugates in plasma/serum samples (53, 54). These conjugates have been generally found to exhibit significantly low cell membrane permeability and thus diminished biological activity (55–57). In contrast, in our differentiation experiments, the phase II conjugates proved to be similarly effective as their parent compounds in promoting osteogenic maturation. One possible explanation is that phase II conjugates may be locally deconjugated by enzymes such as glucuronidases yielding the more bioactive parent compound. Such a deconjugation appears to be enhanced under inflammatory conditions (58–60). As human MSCs have been reported to express the extracellular sulfatase SULF2 (61), one may hypothesize that cell-associated deconjugating enzymes mediate local de-sulfation or deconjugation of phase II metabolites at the cell membrane or in the pericellular matrix, regenerating the active parent compound *in situ*. Thereby, the conjugates would exhibit osteogenic effects comparable to those of free compounds. This remains a hypothesis, since deconjugation activity was not measured in the hMSC cultures of the present study; direct confirmation would require such investigations in future experiments. Indeed, few reports demonstrated that phase II conjugates also exhibit biological activity in a different context (62).

Overall, the results from the hMSC differentiation experiments showing, despite donor-specific effects, a tendency for augmented mineralization are supporting the clinical data from the present study, wherein reduced levels of bone resorption markers were detected. Conventionally, bone health has been solely considered a function of metabolic bone homeostasis, a balanced activity of bone resorption and bone formation. Recent research pointed out that the balance between osteogenesis and adipogenesis plays a crucial role in maintaining bone integrity. Imbalances in this equilibrium favoring development and progression of osteopenia and osteoporosis (12). The present data suggested that Pycnogenol® constituents and metabolites might hold the potential to shift a disrupted differentiation balance towards osteogenic differentiation.

However, the present study has some limitations. First, only women were included in the study, even though participation was open to both sexes. While this may have contributed to consistency of data by increasing homogeneity and eliminating sex-related variability, no direct conclusions can be drawn on possible positive effects of the plant extract in men with low bone mineral density. Nevertheless, the relevance of our findings remains substantial, as postmenopausal women, who represented the majority of participants, are the primary population impacted by osteoporosis (1). In Europe and the US, approximately 30% of all postmenopausal women are estimated to suffer from osteoporosis (63), indicating that the study population reflects the predominant clinical target group. Based on randomized controlled clinical studies with Pycnogenol® comprising more than 2,000 participants, in which no indication of sex-specific effects has been reported (6), comparable responses in men appear plausible. Trials including both sexes would nevertheless be required to address this question directly. Second, the hMSC differentiation experiments were performed with known Pycnogenol® constituents and metabolites instead of using the patients’ serum in the cell culture setting. These serum samples would contain all bioavailable and relevant bioactive molecules and would have more accurately reflected physiological conditions. Using patients’ serum samples before and after intake of the dietary supplement has been previously successfully applied in previous studies (64, 65). For instance, serum obtained from subjects after a single oral dose of 300 mg Pycnogenol® significantly enhanced the inhibition of COX-1 and COX-2 (65) and samples obtained after repeated intake of 200 mg Pycnogenol® significantly inhibited matrix metalloproteinase 9 (MMP-9) release from human monocytes and NF-κB activation (24). Unfortunately, there was insufficient patient serum available in the present study to conduct differentiation experiments following this approach. Third, although reductions in bone resorption markers were observed within the Pycnogenol® group and the *in vitro* experiments indicated enhanced osteogenic differentiation, the underlying molecular and cellular mechanisms were not investigated in more detail and should be addressed in future studies in which larger patient cohorts should be investigated. Recruitment was closed after 67 of the 76 participants originally planned. This met the required number of participants for analysis of CTX-1, the marker on which the main findings are based, but fell below the requirement derived for P1NP, which limits the statistical precision for this parameter. Fourth, CTX-1 concentrations are sensitive to circadian rhythm and food intake. All samples were obtained between 9 a.m. and 1 p.m., at a similar hour for each participant across visits, and participants were instructed to attend in a fasting state, but residual variation in sampling conditions cannot be excluded. Fifth, the short intervention period does not allow for a conclusion regarding the long-term effects. No new fractures occurred during the 6-month observation period, and bone mineral density was assessed at baseline only. Long-term studies designed with fracture incidence or bone mineral density as primary endpoints would be required to establish whether the observed changes in bone turnover markers translate into clinical benefit. The strength of the present study was the experimental design as a randomized placebo-controlled double-blind clinical trial.

To summarize, this investigation was the first randomized placebo-controlled human trial evaluating the effects of Pycnogenol® on bone turnover markers in a population with reduced BMD, providing initial evidence for a potentially favorable effect in terms of mitigating bone resorption without compromising bone remodeling capacity.

## 5 Conclusion

In conclusion, our study demonstrated that Pycnogenol® supplementation was associated with reductions in key bone resorption markers (CTX-1 and NTX-1) within the treatment group *in vivo* while *in vitro* data supported the notion that Pycnogenol® components significantly enhanced the osteogenic differentiation of hMSCs. Saliva concentrations of the gut-derived metabolite 5-(3,4-dihydroxyphenyl)-γ-valerolactone (M1) significantly increased in the Pycnogenol® group compared to placebo at the end of the placebo-controlled phase and the M1 concentrations were inversely correlated with the bone resorption markers CTX-1 and NTX-1. These findings support further investigation of Pycnogenol® in clinical settings as an adjunctive approach in patients with osteopenia or clinically stable osteoporosis.

## Data Availability

The raw data supporting the conclusions of this article will be made available by the authors, without undue reservation.
