# Supplementary material for: Impact of a dietary intervention with French maritime pine bark extract (Pycnogenol®) on bone turnover markers in women with osteoporotic bone loss: a randomized, placebo-controlled double-blind study—Part I
Source: Front Nutr. 2026 Oct 12;13:1923949. doi: 10.3389/fnut.2026.1923949 (PMC13696297; doi:10.3389/fnut.2026.1923949)
Supplement: Supplementary file 1 [file Table_1.docx]

**Supplementary Table S1**: Outliers identified among clinical laboratory parameters. Data excluded due to outliers employing the ROUT method (Q = 1%).

PYC, Pycnogenol^®^, PLAC, placebo

| Parameter | Baseline | | 3 Months | | 6 Months | |
| --- | --- | --- | --- | --- | --- | --- |
|  | PYC | PLAC | PYC | PLAC | PYC | PYC |
| CTX-1 | N = 2 | N = 0 | N = 1 | N = 0 | N = 0 | N = 0 |
| P1NP | N = 2 | N = 0 | N = 2 | N = 0 | N = 1 | N = 1 |
| NTX-1 | N = 0 | N = 0 | N = 0 | N = 0 | N = 1 | N = 0 |
| TRAP5b | N = 0 | N = 1 | N = 0 | N = 1 | N = 0 | N = 1 |
| BSP | N = 2 | N = 2 | N = 3 | N = 4 | N = 1 | N = 2 |
| IL-6 | N =3 | N = 6 | N = 6 | N = 6 | N = 8 | N = 7 |
| M1 | N = 1 | N = 3 | N = 3 | N = 3 | N = 2 | N = 4 |
